# Impact of *PTBP1* rs11085226 on glucose-stimulated insulin release in adult Danes

**DOI:** 10.1186/s12881-015-0160-7

**Published:** 2015-03-20

**Authors:** Tue H Hansen, Henrik Vestergaard, Torben Jørgensen, Marit Eika Jørgensen, Torsten Lauritzen, Ivan Brandslund, Cramer Christensen, Oluf Pedersen, Torben Hansen, Anette P Gjesing

**Affiliations:** The Novo Nordisk Foundation Center for Basic Metabolic Research, Section of Metabolic Genetics, Faculty of Health and Medical Sciences, University of Copenhagen, Universitetsparken 1, DK-2100 Copenhagen, Denmark; Research Centre for Prevention and Health, Glostrup University Hospital, Nordre Ringvej 57, Building 84-85, DK-2600 Glostrup, Denmark; Faculty of Health and Medical Sciences, University of Copenhagen, Copenhagen, Denmark; Steno Diabetes Center, Niels Steensens Vej 2, DK-2820 Gentofte, Denmark; Department of Public Health, Section of General Practice, Aarhus University, Bartholins Allé 2, DK-8000 Aarhus, Denmark; Department of Clinical Biochemistry, Vejle Hospital, Kabbeltoft 25, DK-7100 Vejle, Denmark; Institute of Regional Health Research, University of Southern Denmark, Odense, Denmark; Department of Internal Medicine and Endocrinology, SLB, Vejle Hospital, Kabbeltoft 25, DK-7100 Vejle, Denmark; Faculty of Health Sciences, University of Southern Denmark, Odense, Denmark

**Keywords:** Polypyrimidine tract binding protein 1, Type 2 diabetes, Beta-cell function, Genetics, Insulin release

## Abstract

**Background:**

The variant rs11085226 (G) within the gene encoding polypyrimidine tract binding protein 1 (*PTBP1*) was reported to associate with reduced insulin release determined by an oral glucose tolerance test (OGTT) as well as an intravenous glucose tolerance test (IVGTT). The aim of the present study was to validate the association of the rs11085226 G-allele of *PTBP1* with previously investigated OGTT- and IVGTT-derived diabetes-related metabolic quantitative phenotypes, to conduct exploratory analyses of additional measures of beta-cell function, and to further investigate a potential association with type 2 diabetes.

**Methods:**

*PTBP1* rs11085226 was genotyped in 20,911 individuals of Danish Caucasian ethnicity ascertained from 9 study samples. Case control analysis was performed on 5,634 type 2 diabetic patients and 11,319 individuals having a normal fasting glucose level as well as 4,641 glucose tolerant controls, respectively. Quantitative trait analyses were performed in up to 13,605 individuals subjected to an OGTT or blood samples obtained after an overnight fast, as well as in 596 individuals subjected to an IVGTT.

**Results:**

Analyses of fasting and OGTT-derived quantitative traits did not show any significant associations with the *PTBP1* rs11085226 variant. Meta-analysis of IVGTT-derived quantitative traits showed a nominally significant association between the variant and reduced beta-cell responsiveness to glucose (*β* = −0.1 mmol · kg^−1^ · min^−1^; 95% CI: −0.200.20 – −0.024; *P* = 0.01) assuming a dominant model of inheritance, but failed to replicate a previously reported association with area under the curve (AUC) for insulin. Case control analysis did not show an association of the *PTBP1* rs11085226 variant with type 2 diabetes.

**Conclusions:**

Despite failure to replicate the previously reported associations of *PTBP1* rs11085226 with OGTT- and IVGTT-derived measures of beta-cell function, we did find a nominally significant association with reduced beta-cell responsiveness to glucose during an IVGTT, a trait not previously investigated, leaving the potential influence of this variant in *PTBP1* on glucose stimulated insulin release open for further investigation. However, the present study does not support the hypothesis that the variant confers risk of type 2 diabetes.

**Electronic supplementary material:**

The online version of this article (doi:10.1186/s12881-015-0160-7) contains supplementary material, which is available to authorized users.

## Background

The global prevalence of type 2 diabetes (T2D) is reaching pandemic proportions with an alarming estimate of 439 million affected individuals world-wide (equal to 7.7% of the world’s population) by the year 2030 [1]. It is well established, that the hyperglycemia observed in T2D arises due to a combination of peripheral insulin resistance and impaired pancreatic beta-cell function and consequently reduced insulin secretion [2,3]. T2D is a heritable [4-6], complex metabolic disorder involving several molecular pathways with currently 90 known genetic susceptibility loci, most of which have been identified in recent years by large-scale genome-wide association studies (GWAS) [7]. However, despite recent advances in the understanding of the genetic mechanisms underlying T2D, a substantial part of the heritability (~80-90%) remains unexplained [8].

A candidate-gene study by Heni and colleagues [9] reported a nominal association between reduced glucose stimulated insulin release and the rs11085226 G-allele of the gene encoding polypyrimidine tract binding protein 1 (PTBP1). PTBP1 is a 57 kDa protein consisting of four RNA recognition motifs [10]. It is involved in pre-mRNA and mRNA metabolism as a regulator of alternative splicing, polyadenylation, mRNA stability and initiation of translation [11-13]. It facilitates the biosynthesis and secretion of insulin by binding the pyrimidine rich tract of the 3′-untranslated region of insulin mRNA [14] and other mRNA molecules encoding secretory proteins present in insulin granules of the pancreatic beta-cell [15-17], thus increasing mRNA stability and translation [12,18-20]. Disruption of PTBP1 function, either by siRNA mediated inhibition or mutation of the PTB binding site, results in insulin mRNA destabilization and lower insulin contents [14,15]. Similarly, depletion of PTB levels by microRNA mediated inhibition of PTB-mRNA translation, lowers insulin biosynthesis rates [21]. Also, nuclear retention of PTBP1 has been proposed as a contributing factor in the impairment of rapid glucose-stimulated insulin secretion observed in type 2 diabetic individuals [22]. Variation in the *PTBP1* locus may thus affect insulin secretion and could potentially be associated with a type 2 diabetic phenotype.

Assuming a dominant model of inheritance, Heni et al. [9] found that the minor G-allele of *PTBP1* rs11085226 was nominally associated with a lower insulinogenic index (IGI) and lower corrected insulin response (CIR) in 1,502 healthy individuals of German ethnicity subjected to an oral glucose tolerance test (OGTT). In a subset of participants, the variant was also nominally associated with lower insulin levels within the first ten minutes of an intravenous glucose tolerance test (IVGTT). Three additional tag SNPs (rs351974, rs736926 and rs123698) were examined showing no association with OGTT derived measures and only the rs351974 was associated with decreased insulin secretion measured as the AUC for insulin within the first 10 minutes of an IVGTT. The authors also noted that the rs11085226 variant was nominally associated with reduced homeostatic model assessment of beta-cell function (P = .01815) in the publicly available Meta-Analyses of Glucose and Insulin-related traits (MAGIC) consortium dataset [23].

Thus, the aim of the present study was to validate the association of *PTBP1* rs11085226 with previously investigated OGTT- and IVGTT-derived diabetes-related metabolic quantitative phenotypes, to conduct exploratory analyses of additional measures of beta-cell function, and to further investigate the association with T2D.

## Methods

### Subjects and genotyping

Using the KASP^TM^ di-allelic discrimination (LGC Genomics, Herts, UK), the *PTBP1* rs11085226 was successfully genotyped in 20,821 individuals, ascertained from 9 study groups (Additional file 1: Table A). Discordance between 1,361 random duplicate samples was 0.0%, and the genotyping success rate was 98.1%. The examined marker obeyed Hardy-Weinberg equilibrium (*P* = 0.5) with a minor allele frequency (MAF) of 0.084 (95% CI 0.082 – 0.087) which is close to that reported by the International HapMap project (release 24, November 2010, http://www.hapmap.org) [24] for a population of northern and western European origin (MAF = 0.092; 95% CI 0.055 – 0.131).

Case–control analyses were performed with 5,634 type 2 diabetic individuals (study groups 1–6) and non-diabetic controls consisting of either 4,641 glucose-tolerant (as determined by an OGTT) individuals (study groups 1 & 4), or 11,319 individuals having a normal fasting glucose level (excluding impaired glucose tolerance where OGTT data is available; study groups 1–4,6) (Additional file 1: Table B). Analysis of diabetes-related quantitative traits was performed on 13,605 non-diabetic individuals (study groups 1,2,4,6,7), of which 6,183 (study groups 1,4,7) were subjected to an OGTT, and 596 non-diabetic participants (study groups 6, 11) subjected to an IVGTT (Additional file 1: Table B). All participants were Danish Caucasians by self-report and written informed consent was obtained from all subjects prior to participation. Glucose tolerance status was determined by an OGTT and T2D was defined according to the 1999 World Health Organization criteria [25]. All studies were approved by the Ethical Committee for the County of Copenhagen (study groups 1–4,7-9) or the Region of Southern Denmark (study groups 5 and 6), the Danish Data Protection Agency and conducted in accordance with the Helsinki Declaration. .

### Anthropometrics

In all study groups, body weight and height were measured in light indoor clothes and without shoes. BMI was defined as weight in kilograms divided by height in meters squared (kg/m^2^).

### OGTT

After a 12-h overnight fast, participants in study groups 1 and 4 underwent a standard 75 g OGTT. Serum insulin and plasma glucose was measured at 0, 30 and 120 minutes. Serum insulin levels (excluding des-31,32 and intact proinsulin) were measured using the AutoDELFIA insulin kit (Perkin-Elmer, Wallac, Turku, Finland). Plasma glucose was analyzed using a glucose oxidase method (Granutest; Merck, Darmstadt, Germany) [26].

### IVGTT

#### Youth92

Following a 12-h overnight fast, individuals underwent an IVGTT. After insertion of a cannula into the antecubital vein each subject rested in a quiet room for at least 20 min. Baseline values of serum insulin, serum C-peptide, and plasma glucose were taken in duplicate with 5-min intervals. Glucose was injected intravenously in the contralateral antecubital vein over a period of 60 s (0.3 g/kg body weight of 50% glucose). At 20 min after the glucose injection, a bolus of 3 mg tolbutamide/kg body weight (Rastinon, Hoechst, Germany) was injected over 5 s to elicit a secondary pancreatic beta-cell response. Venous blood was sampled at 2, 4, 8, 19, 22, 30, 40, 50, 70, 90, and 180 min, after glucose injection for measurements of plasma glucose, serum insulin and serum C-peptide. All IVGTTs were done by the same investigator. Plasma concentration of glucose (Diagnostica, Boehringer Mannheim GmbH, Mannheim, Germany) was analyzed using standardized methods. Serum insulin levels (excluding des-31,32 and intact proinsulin) were measured by enzyme-linked immunosorbent assay (ELISA), applying the Dako insulin kit with overnight incubation (code No. K6219; Dako Diagnostics Ltd., Ely, United Kingdom) [27]. The concentration of C-peptide was determined by radioimmunoassay [28] using the polyclonal antibody M1230 [29,30].

#### Family studies

After a 12-hour overnight fast, participants were subjected to an IVGTT in which glucose min (0.3 g/kg body weight of 50% glucose) was injected in the contralateral antecubital vein over a period of 1 minute. At 20 min, a bolus of 3 mg tolbutamide/kg body weight (Orinase, Upjohn, Kalamazoo, MI, USA) was injected over 5 seconds to elicit a secondary pancreatic beta-cell response. Venous blood samples were drawn in triplicate at 2, 3, 4, 5, 6, 7, 8, 10, 12, 14, 16, 19, 22, 23, 24, 25, 27, 30, 35, 40, 50, 60, 70, 80, 90, 100, 120, 140, 160, and 180 minutes for analysis of plasma concentration of glucose and serum insulin. The plasma glucose concentration was analyzed by a glucose oxidase method (Granutest, Merck, Darmstadt, Germany). Serum insulin was determined by ELISA excluding des-31,32 and intact proinsulin [27].

### Calculations and statistical analyses

Statistical analyses were performed using RGui v3.0.1 (http://www.r-project.org/) except the family study sample which was analyzed using the SOLAR software package (http://solar.txbiomedgenetics.org/) taking family relation into account through variance component analysis of multipoint relative-pair identity-by-descent probabilities [31]. Hardy-Weinberg equilibrium was tested using continuity corrected *χ*^2^ test. For all analyses, an uncorrected two-tailed *P*-value <0.05 was considered significant. Individuals with unknown diabetes status were excluded from the analyses.

#### Case control analysis for T2D

To examine differences in genotype distributions between affected and unaffected subjects (either glucose tolerant or having a normal fasting glucose level, categories non-mutually exclusive) logistic regression was applied, adjusting for sex, age, BMI, and study group. Analyses were conducted assuming either an additive or dominant inheritance model. Differences in allele frequency were examined using Fisher’s exact test.

#### Quantitative trait analyses – Fasting/OGTT

Insulinogenic index (IGI) was calculated as (Ins_30_ − Ins_0_)/Glu_30_− Glu_0_) [32]. Corrected insulin response (CIR) was calculated as (100 · Ins_30_)/(Glu_30_ · [Glu_30_ – 3.89]) [33]. The homeostatic model assessment of beta-cell function (HOMA-B) and insulin resistance (HOMA-IR) was calculated as (20 · Ins_0_)/(Glu_0_ – 3.5) and (Ins_0_ · Glu_0_)/22.5 respectively [34]. BIGTT-AIR was calculated according to Hansen et al. [35], insulin sensitivity (ISI) was calculated according to Matsuda et al. [36] and the disposition index (DI) as ISI · IGI. Areas under the curve (AUC) were calculated using the trapezoidal method and incremental values represents the expression above basal values. A general linear model was used to test quantitative traits for differences between genotype groups, excluding individuals with known or screen detected diabetes. Analyses were performed for an additive and dominant model with adjustment for sex, age, study group and BMI with and without adjustment for insulin resistance (HOMA-IR). In a subset of individuals (study group 1) the analysis was adjusted for sex, age, BMI and insulin sensitivity (Matsuda). Analyses of DI were adjusted for age, sex, and BMI only. Logarithmic transformation was applied where appropriate.

#### Quantitative trait analyses – IVGTT

Insulin sensitivity index and glucose effectiveness were calculated using the Bergman MINIMOD computer program developed specifically for the combined intravenous glucose and tolbutamide tolerance test [37-41]. Acute phase insulin (AIR) and C-peptide (ACR) responses were calculated by means of the trapezoidal rule as the incremental values (areas under the curve when expressed above basal values) from 0 to 8 min. Insulin secretion rate (ISR) was estimated from measured serum C-peptide concentrations applying the ISEC software to perform deconvolution [42]. The beta-cell responsiveness to glucose was established by using the linear relationship between ISR and glucose in all participants as an index of beta cell response to glucose (increase in ISR per unit increase in plasma glucose) [43]. The disposition index (DI) was calculated as the product of insulin sensitivity index and first phase insulin responses (0–8 min) [44,45]. Meta-analyses were performed using effect size estimates and SE derived from a linear regression analysis, assuming either an additive or dominant inheritance model with adjustment for age, sex, BMI and insulin sensitivity (Bergman MINIMOD). Analyses of DI were adjusted for age, sex, and BMI only. Traits were log transformed where appropriate. In the meta-analyses both fixed effect (weight of studies estimated using inverse variance) and random effect (weight of studies estimated using DerSimonian-Laird method) [46] were reported.

## Results

### Case control analysis for T2D

Association analysis in 5,634 T2D individuals and 4,641 glucose-tolerant control subjects showed no significant difference in genotype distribution or allele frequency between affected and unaffected individuals for neither an additive or dominant model of inheritance (Table 1). Expanding the control group to 11,319 individuals by including all individuals having normal fasting glycaemia, did not reveal any significant association across inheritance models (Additional file 1: Table C). To address the issue of collinearity arising from inclusion of study groups with T2D individuals or controls only, further analyses in a subset of studies with both outcomes were performed, showing no significant association between the rs11085226 variant and T2D (Additional file 1: Tables D and E).

**Table 1:** **Association analysis of type 2 diabetes and** ***PTBP1*** **rs11085226 in 5,634 type 2 diabetes patients and 4,641 glucose-tolerant control subjects**

	**Genotype distribution**		**Additive model**		**Dominant model**	
	**NGT**	**T2D**	**OR**	***P***	**OR**	***P***
*N*	4641	5634	1.13 (0.88 - 1.46)	0.33^a^	1.14 (0.87 - 1.49)	0.33^a^
AA	3874 (83.5)	4741 (84.1)				
AG	739 (15.9)	848 (15.1)				
GG	28 (0.6)	45 (0.8)				
MAF	8.6 (8.0 – 9.1)	8.3 (7.8 – 8.8)	0.97 (0.88 - 1.07)	0.55^b^		

Data are number of subjects in each genotype group (% of each group) and MAF in % (95% CI). OR (95% CI) and *P*-values for genotype distribution were calculated using logistic regression with adjustment for sex, age, BMI and study group (a). OR (95% CI) and *P*-values for allele frequency were calculated using Fisher’s exact test (b). Analyses were conducted for men and women combined assuming either an additive or dominant inheritance model. NGT: Normal glucose tolerance. T2D: Type 2 diabetes.

### Quantitative trait analyses – Fasting/OGTT

Regression analysis of rs11085226 in up to 13,605 non-diabetic individuals, adjusted for age, sex, BMI and study group, with or without adjustment for insulin resistance (HOMA-IR), and assuming either an additive or dominant model of inheritance, showed no significant association with fasting or OGTT-derived variables of glucose homeostasis and beta-cell function (Table 2). In a subset of 5,031 individuals (study group 1) adjustment for insulin sensitivity (Matsuda) in addition to gender, age, and BMI did not reveal any significant associations for neither an additive nor a dominant model (Additional file 1: Table F).

**Table 2:** **Quantitative metabolic traits in up to 13,605 non-diabetic Danish Caucasian subjects stratified according to genotype**

	***n***	**AA**	**AG**	**GG**	***P***_**add**_	***P***_**dom**_
***Glycated hemoglobin***						
**HbA1c** (%)^¤^	13251 (11071/2099/81)	5.61 (0.41)	5.60 (0.41)	5.56 (0.39)	0.14 (0.15)	0.19 (0.22)
***Plasma glucose***						
**Glu**_0_ (mmol/l)	13599 (11378/2139/82)	5.47 (0.53)	5.48 (0.51)	5.49 (0.55)	0.26 (0.28)	0.23 (0.26)
**Glu**_30_ (mmol/l)^¤^	5522 (4631/860/31)	8.58 (1.70)	8.52 (1.74)	8.66 (1.51)	0.85 (0.76)	0.95 (0.61)
**Glu**_120_ (mmol/l)^¤^	6155 (5180/942/33)	5.96 (1.55)	5.93 (1.50)	5.90 (1.62)	0.90 (0.58)	0.90 (0.60)
**AUC**_Glu (mmol/l·min)_^**¤**^	5510 (4621/858/31)	864.70 (144.86)	863.19 (138.62)	862.84 (121.30)	0.99 (0.65)	0.97 (0.56)
***Serum insulin***						
**Ins**_0_ (pmol/l)^¤^	8944 (7504/1388/52)	40.87 (26.56)	40.17 (25.43)	39.84 (24.64)	0.43 (0.29)	0.41 (0.32)
**Ins**_30_ (pmol/l)^¤^	5342 (4468/844/30)	293.95 (187.55)	287.32 (172.96)	271.67 (133.03)	0.92 (0.99)	0.95 (0.92)
**Ins**_120_ (pmol/l)^¤^	5450 (4570/850/30)	207.13 (198.16)	210.64 (207.43)	188.53 (143.48)	0.55 (0.69)	0.52 (0.64)
**AUC**_Ins_ (pmol/l · min)	5045 (4227/791/27)	27795.2 (17784.9)	27412.34 (17018.2)	26148.89 (12052.43)	0.49 (0.90)	0.51 (0.93)
***Indices of insulin secretion and beta-cell function***						
**DI** ^**¤**^	5031 (4216/788/27)	171.94 (73.12)	171.53 (72.63)	176.99 (86.879)	0.37	0.35
**BIGTT-AIR**	5030 (4215/788/27)	1916.03 (2484.30)	1823.89 (1038.50)	1718.15 (578.42)	0.57 (0.49)	0.58 (0.50)
**CIR** ^**¤**^	5270 (4408/832/30)	872.79 (729.90)	874.15 (731.58)	734.96 (443.06)	0.87 (0.87)	0.91 (0.83)
**HOMA-B** ^**¤**^	8927 (7489/1386/52)	63.75 (48.62)	61.75 (36.81)	62.66 (41.39)	0.96 (0.65)	0.93 (0.75)
**AUC**_Ins(0–30)_ **/AUC**_Glu(0–30)_^**¤**^	5174 (4331/814/29)	23.97 (13.84)	23.66 (13.11)	22.43 (8.91)	0.94 (0.97)	0.89 (0.94)
**IGI** ^¤^	5109 (4276/84/29)	104.38 (117.23)	108.27 (133.32)	81.24 (52.88)	0.67 (0.77)	0.58 (0.69)
**AUC**_C-pep_ **/AUC**_Glu_^¤^	5480 (4597/854/29)	268.18 (79.58)	269.96 (81.90)	256.53 (61.19)	0.39 (0.55)	0.31(0.50)

Data are number of available samples (AA/AG/GG) with means (SD) according to genotype. Traits were tested for normality and log transformation was applied (¤) where appropriate. *P*-values were adjusted for age, sex, BMI, and study group with values in parenthesis further adjusted for insulin sensitivity.

### Quantitative trait analyses – IVGTT

**Figure 1:**
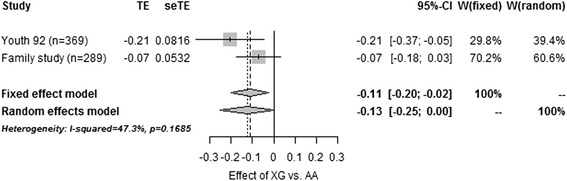
**Forest plot of meta-analysis estimating the combined effect of the G-allele of** ***PTBP1*** **rs11085226 on beta-cell responsiveness to glucose in the Youth92 and Family studies.**

In a fixed-effect meta-analysis of the Family- and Youth 92 study (study groups 8 & 9) including IVGTT-derived traits in 596 individuals, the G-allele was significantly associated with lower beta-cell responsiveness to glucose (*β*-value: −0.11 mmol · kg^−1^ · min^−1^; 95% CI: −0.20 – -0.025; *P* = 0.01) when assuming a dominant model of inheritance and adjusting for age, sex, BMI and insulin sensitivity (Table 3). Despite the absence of any significant between-study heterogeneity (*I*^2^ = 47.3%, *P* = 0.17), only a *P*-value of 0.06 was found when using a random-effect analysis (Figure 1). Assuming an additive model of inheritance neither a fixed nor random effect analysis showed any significant associations with any of the IVGTT-derived traits (Table 3).

**Table 3:** **Meta-analyses estimating the combined effect and 95% confidence interval of the minor allele of** ***PTBP1*** **rs11085226 in the Family- and Youth92 study (** ***n*** **= 596) assuming an additive and dominant model of inheritance**

	**Additive inheritance**				**Dominant inheritance**			
	***Fixed effect model***		***Random effect model***		***Fixed effect model***		***Random effect model***	
	**Combined effect**	***P***	**Combined effect**	***P***	**Combined effect**	***P***	**Combined effect**	***P***
**β-cell responsiveness to glucose** (pmol · kg^−1^ · min^−1^/mmol · l)	0.054 (−0.097; 0.21)	0.5	0.038 (−0.23; 0.30)	0.8	−0.11 (−0.20; −0.025)	0.01	−0.13 (−0.25; 0.003)	0.06
**Fasting ISR** (pmol · kg^−1^ · min^−1^)	0.037 (−0.057; 0.13)	0.4	0.037 (−0.057; 0.13)	0.4	0.65 (−2.62; 3.92)	0.7	0.56 (−4.57; 5.69)	0.8
Disposition index*	−0.11 (−0.302; 0.081)	0.25	0.51 (−0.99; 2.014)	0.5	−0.1030 (−0.299; 0.093)	0.3	0.51(−0.982; 2.011)	0.96
**AUC ISR**_0–8 min_ * (pmol · kg^−1^)	−0.0015 (−0.082; 0.079)	0.97	−0.0015 (−0.082; 0.079)	0.97	−0.060 (−0.16; 0.043)	0.3	−0.081 (−0.27; 0.11)	0.4
**IncAUC ISR**_0–8 min_ (pmol · kg^−1^)	0.003 (−0.097; 0.10)	0.95	0.003 (−0.097; 0.10)	0.95	0.010 (−0.051; 0.072)	0.7	0.010 (−0.051; 0.072)	0.7
**AUC insulin**_0–8 min_ * (pmol/l · min)	−0.043 (−0.15; 0.068)	0.4	−0.043 (−0.15; 0.068)	0.4	−0.094 (−0.29; 0.11)	0.4	−0.094 (−0.29; 0.11)	0.4
**Acute phase insulin response** (pmol/l · min)	−85.40 (−381.20; 210.41)	0.6	−74.47 (−407.35; 258.41)	0.7	16.73 (−215.63; 249.09)	0.9	3.30 (−288.95; 295.55)	0.98
**AUC glucose**_0–8 min_ (mmol/l · min)	0.39 (−1.42; 2.20)	0.7	0.11 (−2.69; 2.91)	0.9	19.82 (−202.28; 241.92)	0.9	−5.020 (−338.55; 328.51)	0.98
**IncAUC glucose**_0–8 min_ (mmol/l · min)	0.36 (−1.2163; 1.9341)	0.66	0.35 (−1.27; 1.96)	0.7	0.02 (−1.42; 1.46)	0.98	0.16(−2.22; 2.55)	0.9
**AUC C-peptide**_0–8 min_* (pmol/l · min)	−0.0019 (−0.063; 0.059)	0.95	−0.0019 (−0.0632; 0.0594)	0.95	−0.0268 (−0.124; 0.0701)	0.6	−0.0336(−0.1582; 0.0910)	0.6
**Acute phase C-peptide response** (pmol/l · min)	7.69 (−582.011; 597.39)	0.98	10.081 (−586.71; 606.87)	0.97	176.92 (−357.81; 711.66)	0.5	176.92 (−357.81; 711.66)	0.5

Data is mean combined effect (95% CI) adjusted for age, sex, BMI, and insulin sensitivity (disposition index adjusted for age, sex, and BMI only). Estimates of traits displaying non-normality (*) were based on log transformed values. Fixed effect model represent the combined effects of the studies weight using inverse variance. The Random effects model represent the combined effects of the studies weighted using the DerSimonian-Laird method.

## Discussion

In the present study, we tried to replicate the previously reported association of *PTBP1* rs11085226 with reduced glucose stimulated insulin release in a Danish Caucasian population and to complement with an investigation of measures of beta-cell function and the potential association to T2D. Adjusting for the same covariates as in the original study, we were unable to replicate the reported association with OGTT-derived measures of insulin release. Interestingly, in the MAGIC consortium data released in May 2014, the rs11085226 variant was neither associated with DI (P = 0.71), ISI adjusted CIR (P = 0.86) nor any other OGTT-derived indices of glucose-stimulated insulin secretion in up to 5,318 non-diabetic participants from 9 cohorts [47].

In the meta-analysis of IVGTT-derived traits, we did not find an association with AUC insulin, which was previously reported to be significantly reduced in carriers of the rs11085226 minor allele. We did find, however, that the variant is associated with reduced beta-cell responsiveness to glucose which represents the increase in insulin release rate per unit increase in plasma glucose. Interestingly, this measure of acute phase insulin release has previously been found to be reduced by a factor 3 in seven T2D patients of Caucasian ethnicity [43]. In the same study, the reduced beta-cell responsiveness to glucose in T2D patients was rectified during an infusion of a low dose of glucagon-like peptide-1, which points to the possibility that the incretin response following an oral glucose load may compensate for any effect of the rs11085226 G-allele on OGTT-derived measure of beta-cell function. In the present study we did, however, not find an association of the rs11085226 variant with the type 2 diabetic phenotype. According to the database of genetic association studies (available at www.gwascentral.com) the rs11085226 variant has not been tested for association to T2D in individuals of European ancestry, nor is it included in the stage 1 & 2 meta-analysis data of the DIAbetes Genetics Replication And Meta-analysis (DIAGRAM) consortium (data available online at www.diagram-consortium.org) [11]. However, following the completion of our analyses, data from a trans-ethnic meta-analysis including cohorts of individuals with European, Mexican/Mexican American, south Asian and east Asian ancestry became publically available [48], including data on the rs11085226 variant showing no significant association with T2D (OR = 1.01, 95CI: 0.94 - 1.07; *P* = 0.85; *N* = 27767).

Given the limited number of studies included in the meta-analysis, caution should be taken when assessing these results, emphasized by the fact that a random effects analysis did not show a significant effect. It should also be noted that assessment of population stratification as a potential bias in the analyses of IVGTT traits was not possible due to the lack of array based genotype data. Yet, beta-cell responsiveness to glucose is a more accurate measure of insulin release than traits derived from levels of circulating insulin. Obtaining a significant result, even if nominal only, despite the relatively small number of subjects in the present study might emphasize the importance of collecting refined traits when assessing complex physiological processes such as beta-cell function. Similarly, given the limited number of subjects in the present study, it is possible that a small effect of the rs11085226 variant on T2D susceptibility remains undetected due to a lack of statistical power. Furthermore, being a heterogeneous disorder poorly defined by a dichotomized end-point, the variant may only affect disease susceptibility in a subset of type 2 diabetic patients.

Clearly the reported association does not hold for stringent correction for 54 independent tests *ad modum* Bonferroni. However, considering the correlation between the tested traits and the *a priori* knowledge of the involvement of PTBP1 in insulin secretion, applying the Bonferroni correction might be overly conservative. Furthermore, given that the association of the rs11085226 variant with beta-cell responsiveness to glucose has not previously been reported, this part of the analysis is exploratory and we therefore consider it relevant to report significance at the nominal level only.

In the present study, as well as the previous study by Heni and colleagues [9], the association of the rs11085226 G-allele with reduced glucose-stimulated insulin release was found under the assumption of a dominant model of inheritance. PTBP1 exists in solution as a dimer [49], which could potentially explain the genetic dominance. However, neither rs11085226 nor any of its five known proxies (rs10426084, rs351977, rs10422347, rs10420407, and rs10420953 [in LD with *r*^2^ > 0.8 as determined by the Broad Institute's SNP Annotation and Proxy Search website using the CEU panel from the 1000 genomes project pilot 1 and HapMap release 22]), of which only rs10420953 is a coding variant (N108N), results in any non-synonymous amino acid substitutions in the PTBP1 protein. These are however all common variants (MAF > 5%) and one could speculate that the causal variant, which could very well be coding, is rare and uncaptured by chip-based genotyping. Furthermore, a reason why modelling the effect of the variant as additive does not reveal an association might the low number of rare homozygous individuals in the IVGTT cohorts, in which case an additive model does not give an accurate representation of the data.

## Conclusion

Although failing to replicate the previously reported association of *PTBP1* rs11085226 to OGTT-derived mea sures of beta-cell function, we show a nominal significant association of the variant to reduced beta-cell responsiveness to glucose, a measure of glucose stimulated insulin release not previously investigated in relation to *PTBP1*. However, any effect the variant may have on beta-cell function does not appear to have a diabetogenic impact. Larger studies of IVGTT-derived measures of dynamic beta-cell function or meta-analysis thereof are needed to thoroughly investigate a potential effect of the rs11085226 variant on glucose-stimulated insulin release.

## Additional file

Additional file 1:
**Supplementary tables and description of methods.**

## Abbreviations

OGTT: Oral glucose tolerance test
IVGTT: Intravenous glucose tolerance test
ISR: Insulin secretion rate
IGI: Insulinogenic index
IFG: Impaired fasting glucose
AUC: Area under the curve
SNP: Single nucleotide polymorphism
HOMA-B: Homeostatic model assessment of beta-cell function
HOMA-IR: Homeostatic model assessment of insulin resistance
CIR: Corrected insulin response
DI: Disposition index
MAF: Minor allele frequency
PTBP1: Polypyrimidine tract binding protein 1
T2D: Type 2 Diabetes
