# Supplementary material for: Impact of PTBP1 rs11085226 on glucose-stimulated insulin release in adult Danes
Source: BMC Med Genet. 2015 Mar 20;16:17. doi: 10.1186/s12881-015-0160-7 (PMC4422140; doi:10.1186/s12881-015-0160-7)
Supplement: Additional file 1: — Supplementary tables and description of methods. [file 12881_2015_160_MOESM1_ESM.docx]

**Methods**

*ISEC*

Direct measurement of the rate of insulin secretion requires catheterisation of the portal vein and is therefore not suitable for typical clinical conditions. Indirect estimation employing plasma insulin concentration fails due to large and variable hepatic extraction of insulin. C-peptide is not cleared by the liver but the plasma concentration is not sufficient to estimate appearance without the knowledge of the disappearance and vice versa. ISEC determines the C-peptide disappearance model (kinetic model) from subject's individual data [[1](#_ENREF_1)]. The determination is based on a population-based study [[2](#_ENREF_2)] which has shown that the parameters of the C-peptide kinetic model can be approximated from the subject's weight, height, age, sex and classification (normal, obese, NIDDM). However, due to the long half-life of C-peptide, it is difficult to measure fast changes in insulin secretion from peripheral C-peptide[[3](#_ENREF_3)] as the plasma concentration at any given time results from the accumulation of C-peptide in plasma due to immediate and past secretion. This accumulation process is usually denoted by the mathematical term 'convolution'. To reconstruct secretion from plasma concentration, an inverse operation has to be adopted. Thus, ISEC employs a method of deconvolution constrained to non-negative values in order estimate minor changes in secretion [[1](#_ENREF_1)].

*Beta cell responsiveness*

The beta cell responsiveness describes how much the insulin secretion rate (ISR) increases per unit increase in plasma glucose. This estimate is calculated from the slope of the relation between ISR and glucose and is possible due to the linear relation between glucose and ISR as identified by Kjems and colleagues for normal glucose tolerant individuals as well as type 2 diabetes patients [[4](#_ENREF_4)].

**
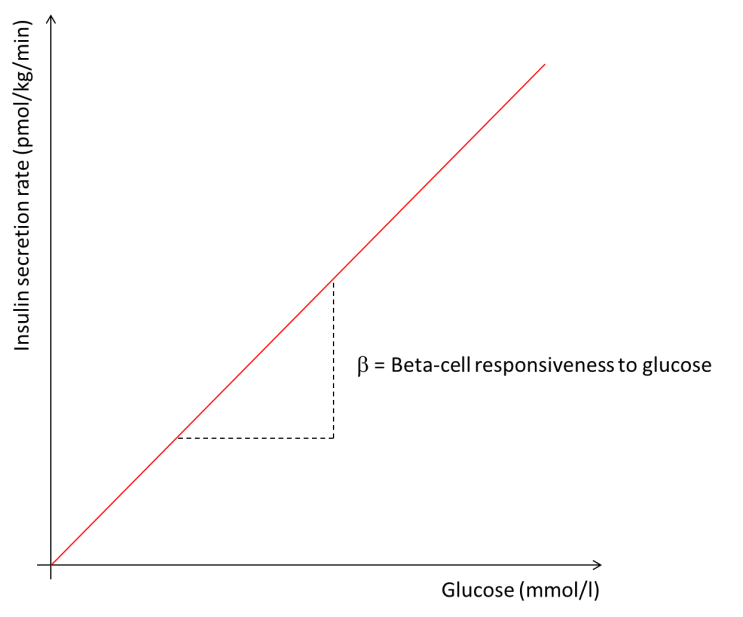
**

**Supplementary tables**

| **Supplementary table A.** Study samples | | | | |
| --- | --- | --- | --- | --- |
| **Study group** | ***n***  (M/F) | **Age**  (Years) | **BMI**  (kg/m^2^) | **Sample Description** |
| **1 *Inter99*** | 6269  (3066/3203) | 46.1  (7.9) | 26.3  (4.6) | DNA-samples from a population-based, randomized, non-pharmacological intervention study of middle-aged individuals for the prevention of ischemic heart disease, conducted at the Research Centre for Prevention and Health, Glostrup, Denmark (ClinicalTrials.gov ID: NCT00289237; Ethical approval ID: KA98155 )[[5](#_ENREF_5)]. |
| **2 *Health 2006*** | 3351  (1497/1854) | 49.3  (13.0) | 25.9  (4.7) | DNA-samples from a general population-based study of individuals aged 18-69 yr (*n* = 3,471) obtained from a random sample of the Danish Civil Registration System. Participants were subjected to a general health examination at the Research Centre for Prevention and Health, Glostrup, Denmark (ClinicalTrials.gov ID: NCT00316667; Ethical approval ID: KA20060011)[[6](#_ENREF_6)]. |
| **3 *SDC T2D*** | 799  (494/305) | 58.6  (10.4) | 29.4  (5.2) | Danish Caucasian type 2 diabetic patients sampled at the outpatient clinic at Steno Diabetes Center, Gentofte, Denmark  (Ethical approval ID: KA96117g). |
| **4 *SDC Control*** | 244  (119/125) | 52.9  (14.0) | 25.7  (4.2) | A population-based sample of healthy Danish Caucasian individuals 30-70 years of age who were recruited at random from the Danish Civil Registration System and examined at Steno Diabetes Center, Gentofte, Denmark (Ethical approval ID: KA94092g). |
| **5 *ADDITION*** | 1608  (916/692) | 60.2  (6.8) | 31.1  (5.4) | DNA-samples from the Anglo-Danish-Dutch Study of Intensive Treatment in People with Screen-Detected Diabetes in Primary Care (ADDITION) Denmark cohort, a population-based, high-risk screening and intervention study for type 2 diabetes in general practice, conducted at the Dept. of General Practice, University of Aarhus, Denmark (ClinicalTrials.gov ID: NCT00237548; Ethical approval ID: 20000183) [[7](#_ENREF_7)]. |
| **6 *Vejle Biobank*** | 7693  (4384/3309) | 60.9  (10.4) | 28.3  (5.4) | DNA-samples from type 2 diabetic patients and matched controls recruited from Vejle Hospital with the intent to investigate the effect of genetic variability on the development of late diabetic complications and lack of response to therapy in type 2 diabetes(Ethical approval ID: S-20080097). |
| **7  *1936 Birth cohort*** | 351  (168/183) | 60.5  (0.4) | 26.8  (4.1) | Danish Caucasian subjects born in 1936 examined at age 60 as part of the 1936 Birth Cohort (all subjects previously examined at age 40 and 45, with male participants undergoing further examination at age 51)(Ethical approval ID: KA96008)[[8](#_ENREF_8)]. |
| **8  *Family cohort*** | 227  (99/128) | 42.28  (11.8) | 26.1  (4.5) | Relatives of patients with verified type 2 diabetes having four or more offspring and a spouse without known diabetes identified through the outpatient clinic at Steno Diabetes Center or through an ongoing family study at the University of Copenhagen (Ethical approval ID: KA93033). |
| **9  *Youth 92*** | 369  (184/185) | 25.2  (3.5) | 23.6  (3.7) | A population-based sample of non-diabetic individuals aged 18–32 yrs, who had previously, as children, participated in blood pressure surveys as part of the Oesterbro study (Ethical approval ID: KA91248)[[9](#_ENREF_9)]. |
| Data is number of genotyped individuals (male/female), age in years (SD) and BMI in kg/m^2^ (SD). | | | | |

| **Supplementary table B.** Genotype distribution and number of subjects according to study material and analysis category | | | | | |
| --- | --- | --- | --- | --- | --- |
| **Study group** | **Case-control analysis** | | | **Quantitative traits analysis** | |
|  | **T2D** | **NGT** | **NFG** | **Fasting + OGTT** | **IVGTT** |
| **1 *Inter99*** | 325  (273/49/3) | 4432  (3690/714/28) | 4691  (3911/750/30) | 5633  (4722/879/32) |  |
| **2 *Health 2006*** | 190  (155/34/1) |  | 2714  (2254/442/18) | 2963  (2468/475/20) |  |
| **3 *SDC T2D*** | 616  (530/86/0) |  | 10  (8/2/0) |  |  |
| **4 *SDC control*** | 19  (11/8/0) | 209  (184/25/0) | 209  (184/25/0) | 224  (196/28/0) |  |
| **5 *ADDITION*** | 1623  (1386/224/13) |  |  |  |  |
| **6 *Vejle biobank*** | 2861  (2386/447/28) |  | 3695  (3080/592/23) | 4459  (3711/719/29) |  |
| **7 *1936 Birth cohort*** |  |  |  | 326  (286/39/1) |  |
| **8 *Family cohort*** |  |  |  |  | 227  (192/33/2) |
| **9 *Youth 92*** |  |  |  |  | 369  (300/68/1) |
|  |  |  |  |  |  |
| **Total** | 5634  (4741/848/45) | 4641  (3874/739/28) | 11319  (9437/1811/71) | 13605  (11383/2140/82) | 596  (492/101/3) |
| Data is number of genotyped individuals (*n*_AA_/*n*_AG_/*n*_GG_) within each study sample and according to analysis category. OGTT: Oral glucose tolerance test. IVGTT: Intravenous glucose tolerance test. T2D: Type 2 diabetes. NGT: Normal glucose tolerance as assessed by an OGTT. NFG: Normal fasting glycemia, excluding impaired glucose tolerance where data from an OGTT is available. The NGT and NFG categories are otherwise non-mutually exclusive. | | | | | |

| **Supplementary table C.** Association studies of type 2 diabetes and *PTBP1* rs11085226 in 5’732 type 2 diabetes patients and 11´941 control subjects (study groups 1-6) with normal fasting glycaemia. | | | | | | |
| --- | --- | --- | --- | --- | --- | --- |
|  | **Genotype Distribution** | | **Additive Model** | | **Dominant Model** | |
|  | **NFG** | **T2D** | **OR** | ***P*_Add_** | **OR** | ***P*_Dom_** |
| *n* | 11319 | 5634 | 0.99  (0.90 - 1.09) | 0.83^a^ | 0.98  (0.88 - 1.09) | 0.67^a^ |
| AA | 9437 (83.4) | 4741 (84.1) |  |  |  |  |
| AG | 1811 (16.0) | 848 (15.1) |  |  |  |  |
| GG | 71 (0.6) | 45 (0.8) |  |  |  |  |
| MAF | 8.6 (8.3 – 9.0) | 8.3 (7.8 – 8.8) | 0.96  (0.89 - 1.04) | 0.35^b^ |  |  |
|  | | | | | | |

| **Supplementary table D.** Association analysis of type 2 diabetes and *PTBP1* rs11085226 in 4,011 type 2 diabetes patients and 11,319 control subjects (study groups 1-4 & 6) with normal fasting glycemia. | | | | | | |
| --- | --- | --- | --- | --- | --- | --- |
|  | **Genotype Distribution** | | **Additive Model** | | **Dominant Model** | |
|  | **NFG** | **T2D** | **OR** | ***P*_Add_** | **OR** | ***P*_Dom_** |
| *N* | 11319 | 4011 | 1.03  (0.93 - 1.15) | 0.58^a^ | 1.02  (0.91 - 1.15) | 0.68^a^ |
| AA | 9437 (83.4) | 3355 (83.6) |  |  |  |  |
| AG | 1811 (16.0) | 624 (15.6) |  |  |  |  |
| GG | 71 (0.6) | 32 (0.8) |  |  |  |  |
| MAF | 8.6 (8.3 – 9.0) | 8.6 (8.0 – 9.2) | 0.99  (0.91 - 1.09) | 0.91^b^ |  |  |
|  | | | | | | |

| **Supplementary table E.** Association analysis of type 2 diabetes and *PTBP1* rs11085226 in 344 type 2 diabetes patients and 4,641 glucose-tolerant control subjects (study groups1 & 4). | | | | | | |
| --- | --- | --- | --- | --- | --- | --- |
|  | **Genotype Distribution** | | **Additive Model** | | **Dominant Model** | |
|  | **NGT** | **T2D** | **OR** | ***P*_Add_** | **OR** | ***P*_Dom_** |
| *N* | 4641 | 344 | 1.21  (0.90 - 1.62) | 0.21^a^ | 1.21  (0.89 - 1.65) | 0.23^a^ |
| AA | 3874 (83.5) | 284 (82.6) |  |  |  |  |
| AG | 739 (15.9) | 57 (16.6) |  |  |  |  |
| GG | 28 (0.6) | 3 (0.9) |  |  |  |  |
| MAF | 8.6 (8.0 – 9.1) | 9.2 (7.0 – 11.3) | 1.08  (0.81 - 1.41) | 0.57^b^ |  |  |
| Data are number of subjects in each genotype group (% of each group) and MAF in % (95% CI). OR (95% CI) and *P*-values for genotype distribution were calculated using logistic regression with adjustment for sex, age, BMI and study group (a). *P*-values for allele frequency were calculated using Fisher’s exact test (b). Analyses were conducted assuming either an additive or dominant inheritance model. NGT: Normal glucose tolerance. NFG: Normal fasting glycaemia. T2D: Type 2 diabetes | | | | | | |

| **Supplementary table F.** Quantitative metabolic traits in the population-based Inter99 cohort including up to 5’031 non-diabetic Danish Caucasian subjects stratified according to genotype. | | | | | | | | | | |
| --- | --- | --- | --- | --- | --- | --- | --- | --- | --- | --- |
|  | ***n*** | **AA** | **AG** | | **GG** | | | ***P***_Add_ | | ***P***_Dom_ |
| ***Glycated hemoglobin*** | | | | | | | | | | |
| **HbA1c** (%)^¤^ | 5025  (4210/788/27)  (4210) | 5.80  (0.40) | 5.79  (0.40) | 5.62  (0.45 | | 0.43 | | | 0.73 | |
| ***Plasma glucose*** | | | | | | | | | | |
| **Glu**_0_ (mmol/l) | 5031  (4216/788/27) | 5.46  (0.52) | 5.45  (0.46) | 5.36  (0.56) | | | 0.76 | | 0.93 | |
| **Glu**_30_ (mmol/l)^¤^ | 5031  (4216/788/27) | 8.59  (1.69) | 8.50  (1.71) | 8.72  (1.36) | | | 0.57 | | 0.46 | |
| **Glu**_120_ (mmol/l)^¤^ | 5031  (4216/788/27) | 6.00  (1.53) | 5.96  (1.47) | 6.06  (1.50) | | | 0.75 | | 0.73 | |
| **AUC**_Glu_ (mmol/l·min)**^¤^** | 5031  (4216/788/27) | 867.52  (144.93) | 859.81  (142.69) | 876.11  (113.70) | | | 0.98 | | 0.82 | |
| ***Serum insulin*** | | | | | | | | | | |
| **Ins**_0_ (pmol/l)^¤^ | 5031  (4216/788/27) | 41.29  (26.49) | 41.09  (26.30) | 39.78  (22.07) | | | 0.64 | | 0.58 | |
| **Ins**_30_ (pmol/l)^¤^ | 5031  (4216/788/27) | 296.17  (189.95) | 289.48  (175.01) | 278.93  (134.75) | | | 0.5 | | 0.49 | |
| **Ins**_120_ (pmol/l)^¤^ | 5031  (4216/788/27) | 208.85  (199.99) | 210.09  (196.10) | 195.93  (149.51) | | | 0.98 | | 0.84 | |
| **AUC**_Ins_ (pmol/l·min) | 5031  (4216/788/27) | 27787.89  (17783.07) | 27439.16  (17040.90) | 26148.89  (12052.43) | | | 0.77 | | 0.82 | |
| ***Indices of insulin secretion and cell function*** | | | | | | | | | | |
| **DI**^¤^ | 5025  (4210/788/27) | 171.94  (73.12) | 171.53  (72.63) | 176.99  (86.87) | | | 0.63 | | 0.58 | |
| **BIGTT-AIR** | 5030  (4215/788/27) | 1916.03  (2484.30) | 1823.89  (1038.50) | 1718.15  (578.42) | | | 0.49 | | 0.5 | |
| **CIR^¤^** | 4974  (4169/788/27) | 877.49  (720.41) | 892.64  (746.62) | 715.06  (381.09) | | | 0.97 | | 0.93 | |
| **AUC**_Ins(0-30)_**/AUC**_Glu (0-30)_^¤^ | 5031  (4216/788/27) | 24.08  (13.94) | 23.80  (13.21) | 22.51  (8.97) | | | 0.65 | | 0.66 | |
| **HOMA-B^¤^** | 5026  (4211/788/27) | 62.78  (47.33) | 62.35  (39.63) | 63.21  (29.75) | | | 0.73 | | 0.83 | |
| **AUC**_C-pep_ **/AUC**_Glu_^¤^ | 5030  (4215/788/27) | 269.18  (80.12) | 271.29  (82.51) | 254.52  (58.83) | | | 0.53 | | 0.46 | |
| **IGI**^¤^ | 4968  (4163/788/27 | 105.10  (118.35) | 109.36  (135.19) | 77.48  (44.60) | | | 0.81 | | 0.62 | |
| Data are number of available samples (AA/AG/GG) with means (SD) according to genotype. Traits were tested for normality and log transformation was applied (¤) where appropriate. *P*-values were adjusted for age, sex, BMI, and insulin sensitivity (disposition index adjusted for age, sex, and BMI only). | | | | | | | | | | |

**Supplementary figure A.** Principal component analysis of population structure in study groups 1-6 based on HumanExome BeadChip genotype data.


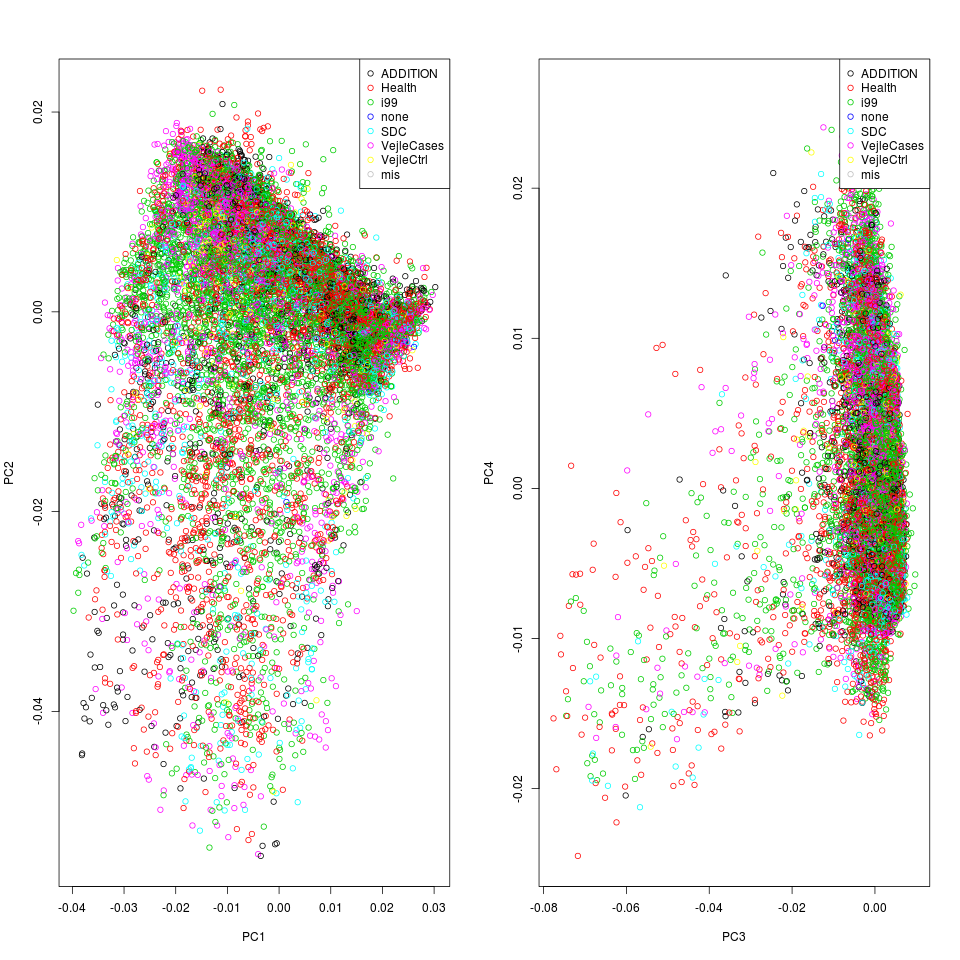


**References**

1. Hovorka R, Soons PA, Young MA: **ISEC: a program to calculate insulin secretion**. *Computer Methods and Programs in Biomedicine* 1996, **50**(3):253-264.

2. Van Cauter E, Mestrez F, Sturis J, Polonsky KS: **Estimation of insulin secretion rates from C-peptide levels. Comparison of individual and standard kinetic parameters for C-peptide clearance**. *Diabetes* 1992, **41**(3):368-377.

3. Kjems LL, Christiansen E, Volund A, Bergman RN, Madsbad S: **Validation of methods for measurement of insulin secretion in humans in vivo**. *Diabetes* 2000, **49**(4):580-588.

4. Kjems LL, Holst JJ, Volund A, Madsbad S: **The influence of GLP-1 on glucose-stimulated insulin secretion: effects on beta-cell sensitivity in type 2 and nondiabetic subjects**. *Diabetes* 2003, **52**(2):380-386.

5. Jorgensen T, Borch-Johnsen K, Thomsen TF, Ibsen H, Glumer C, Pisinger C: **A randomized non-pharmacological intervention study for prevention of ischaemic heart disease: baseline results Inter99**. *European journal of cardiovascular prevention and rehabilitation : official journal of the European Society of Cardiology, Working Groups on Epidemiology & Prevention and Cardiac Rehabilitation and Exercise Physiology* 2003, **10**(5):377-386.

6. Thuesen BH, Cerqueira C, Aadahl M, Ebstrup JF, Toft U, Thyssen JP, Fenger RV, Hersoug LG, Elberling J, Pedersen O *et al*: **Cohort Profile: The Health2006 cohort, Research Centre for Prevention and Health**. *International journal of epidemiology* 2013.

7. Lauritzen T, Griffin S, Borch-Johnsen K, Wareham NJ, Wolffenbuttel BH, Rutten G: **The ADDITION study: proposed trial of the cost-effectiveness of an intensive multifactorial intervention on morbidity and mortality among people with Type 2 diabetes detected by screening**. *International journal of obesity and related metabolic disorders : journal of the International Association for the Study of Obesity* 2000, **24 Suppl 3**:S6-11.

8. Drivsholm T, Ibsen H, Schroll M, Davidsen M, Borch-Johnsen K: **Increasing prevalence of diabetes mellitus and impaired glucose tolerance among 60-year-old Danes**. *Diabetic medicine : a journal of the British Diabetic Association* 2001, **18**(2):126-132.

9. Clausen JO, Borch-Johnsen K, Ibsen H, Bergman RN, Hougaard P, Winther K, Pedersen O: **Insulin sensitivity index, acute insulin response, and glucose effectiveness in a population-based sample of 380 young healthy Caucasians. Analysis of the impact of gender, body fat, physical fitness, and life-style factors**. *The Journal of Clinical Investigation* 1996, **98**(5):1195-1209.
